# Nitrogen-doped tungsten carbide nanoarray as an efficient bifunctional electrocatalyst for water splitting in acid

**DOI:** 10.1038/s41467-018-03429-z

**Published:** 2018-03-02

**Authors:** Nana Han, Ke R. Yang, Zhiyi Lu, Yingjie Li, Wenwen Xu, Tengfei Gao, Zhao Cai, Ying Zhang, Victor S. Batista, Wen Liu, Xiaoming Sun

**Affiliations:** 1 State Key Laboratory of Chemical Resource Engineering, College of Energy, and Beijing Advanced Innovation Centre for Soft Matter Science and Engineering, Beijing University of Chemical Technology, 100029 Beijing, China; 2 Department of Chemistry and Energy Sciences Institute, Yale University, 810 West Campus Drive, West Haven, CT 06516 USA

## Abstract

Tungsten carbide is one of the most promising electrocatalysts for the hydrogen evolution reaction, although it exhibits sluggish kinetics due to a strong tungsten-hydrogen bond. In addition, tungsten carbide’s catalytic activity toward the oxygen evolution reaction has yet to be reported. Here, we introduce a superaerophobic nitrogen-doped tungsten carbide nanoarray electrode exhibiting high stability and activity toward hydrogen evolution reaction as well as driving oxygen evolution efficiently in acid. Nitrogen-doping and nanoarray structure accelerate hydrogen gas release from the electrode, realizing a current density of −200 mA cm^−2^ at the potential of −190 mV vs. reversible hydrogen electrode, which manifest one of the best non-noble metal catalysts for hydrogen evolution reaction. Under acidic conditions (0.5 M sulfuric acid), water splitting catalyzed by nitrogen-doped tungsten carbide nanoarray starts from about 1.4 V, and outperforms most other water splitting catalysts.

## Abstract (web summary)

Water electrolysis can generate carbon-neutral hydrogen gas from water, yet the required catalysts are often expensive, scarce, and poor at gas release. Here, the authors prepared nitrogen-doped carbon tungstide nanoarrays with high water-splitting activities and bubble-releasing surfaces.

## Introduction

Hydrogen gas has long been regarded as a clean and sustainable energy carrier for replacing traditional fossil fuels. Cost-effective manufacture of hydrogen gas is one of the key points that remain to be addressed for a successful hydrogen economy^1–3^. Water electrolysis driven by electricity from sustainable energy sources (e.g., wind, or solar) is an environmentally friendly scheme to produce hydrogen gas with high purity^4–11^. Compared with alkaline electrolysis, hydrogen production in acids like in polymer electrolyte membrane electrolysis has the advantages of simplicity, high-current density, and high-pressure compatibility. However, high-performance catalysts for HER and OER in acid media are mostly limited to noble metals (e.g., platinum (Pt), iridium (Ir), and ruthenium (Ru)). The expensive cost and limited reserve of noble metals have restricted their wide application. Remarkable endeavors have been made to develop non-noble metal catalysts for HER in acid^12^, such as metal sulfides^13–17^, metal carbides^5,18–20^, and metal phosphide^21–23^. Unfortunately, most non-noble metal catalysts for HER in acid are either of large onset overpotential^24–27^ or lack of working stability especially at high current densities^11,16–19^. As for OER in acid electrolyte, the effective catalysts are still limited to noble metal catalysts^28,29^, while only a few non-noble metal catalysts (such as Co_3_O_4_^30^, MnO_x_^31^, and CoMnO_x_^32^) have been reported, but with large onset potentials and poor stability.

Tungsten carbide (WC) is an earth-abundant and inexpensive catalyst, which is stable in acid and behaves like Pt in hydrogenolysis due to its electron configuration around the Fermi level^33,34^. These properties make WC a promising candidate for applications as a non-noble metal electrocatalyst for HER in acid^5,18,35,36^. Despite its popularity, our theoretical calculations show that adsorbed hydrogen atoms bind strongly to WC favoring H^+^ reduction but hindering hydrogen desorption. It is therefore important to explore materials based on WC with better performance. Tungsten carbide nanorods have been synthesized without template^37,38^. In 2013, Hashimoto et al. synthesized a Fe-WCN electrocatalyst^39^. They suggested that the electron density of W atoms was abated by W–N bond and therefore improved the HER catalytic activity. Next, Chen et al. reported an electrocatalyst that combined tungsten carbide-nitride and graphene nanoplatelets, showing that synergistic effects between W_2_C and WN phases promote outstanding hydrogen evolution catalytic activity^40^. However, the effect was limited to only low current densities and the underlying mechanisms remained ambiguous. As a result, none of the materials displayed current densities as large as −200 mA cm^−2^. Furthermore, besides the catalyst’s intrinsic activity, gas bubbles’ fast removal from the surface of the electrode is important for efficient gas evolution. Strong bubble adherence to the electrode surface interferes with charge and mass transfer between the catalyst and the electrolyte. Catalysts may also be peeled off upon departure of bubbles due to strong adhesion. Therefore, the activity of catalysts for gas evolution reactions can be significantly improved with superaerophobic electrodes^20,21^. Electrode design based on nanostructuring and atomic level modulation is thus a promising approach to accelerate the activity and stability for hydrogen evolution reaction.

Here, we report a superaerophobic catalytic electrode for both HER and OER in acid based on N-doped WC nanoarray structures. The design strategy is multifold. N-doping modulates the surface energy level to optimize hydrogen binding and thus promote HER kinetics. The nanoarray structure not only exposes more active sites for electrochemical reaction but also facilitates gas release by offering a superaerophobic interface under water. Therefore, the interface has weak bubble adhesion, detaching small bubbles with little contact area that favors gas evolution at heterogeneous catalytic surfaces. As expected, the N-WC nanoarray electrode offers excellent HER activity and stability to −200 mA cm^−2^, outperforming the vast majority of reported non-precious metal electrocatalysts. Moreover, the N-WC nanoarray also shows excellent overall water splitting performance in acid, functioning as both cathode and anode with reasonable stability.

## Results

### Theoretical calculations

**Fig. 1:**
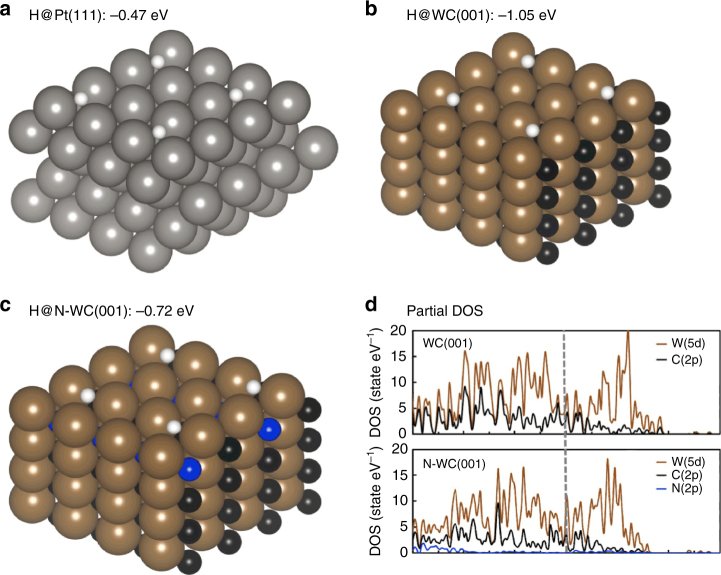
DFT hydrogen binding geometries and binding energies. Hydrogen binding geometries and binding energies of **a** WC (001) surface, **b** N-WC (001) surface, **c** Pt (111) surface, and **d** calculated partial density of state (DOS) of WC (001) and N-WC (001). The vertical dash line denotes the position of the Fermi level. Color key: black, brown, white, blue, and gray balls represent C, W, H, N, and Pt atoms, respectively

Density functional theory (DFT) calculations were performed to study the binding of hydrogen with different catalytic surfaces, and correlated the calculated hydrogen binding energies (HBE) to HER activity. The calculation result of HBE on Pt (111) is −0.47 eV, as shown in Fig. 1a, which is quite similar to the reported result of −0.46 eV^41^, confirming the validity of our calculation method. We further focus on the hydrogen binding to the (001) surface of WC since it has been shown to be the most stable surface, according to an earlier study^42^. As displayed in Fig. 1b, hydrogen atoms bind strongly in between two W atoms, with a calculated HBE per H atom of −1.05 eV which is significantly more negative than that for Pt (111) (−0.47 eV). Therefore, the surface bound hydrogen atoms do not come off the surface so easily as H_2_ from the WC (001) surface, consistent with the observed low HER activity of WC. Remarkably, the HBE on N-WC (001) surface decreases to −0.72 eV (Fig. 1c), upon doping WC with N, becoming much closer to H bonding on Pt (111). Therefore, the calculated HBEs suggest that the HER activity of WC can be improved significantly through N-doping. The variation of HBEs with different N-doping amount has also been investigated, and demonstrated 6.25 at.% of N-doping in N-WC as the optimum value (Supplementary Fig. 1).

The partial density of states (DOS) on individual surface atoms of WC are displayed in Fig. 1d, as compared to those in N-WC, to analyze the N-doping effect on WC’s electronic structure. We note that the density of 5d states of W atoms on N-WC (001) surfaces is downshift relative to that on WC (001) surfaces, suggesting a more negative d-band center of W atoms on N-WC (001). Indeed, the d-band center of W on WC (001) is calculated to be −1.12 eV, while that on N-WC (001) is calculated to be −1.50 eV, close to that on Pt (111) surfaces. The lower d-band center results in weaker H adsorption to the catalytic surface (Supplementary Fig. 2)^43^. These results provide valuable insights on N-doping effect on the electronic structure, and imply more suitable H adsorption on the N-WC surface for promoting HER activity.

### Synthesis of N-WC nanoarray electrode

**Fig. 2:**
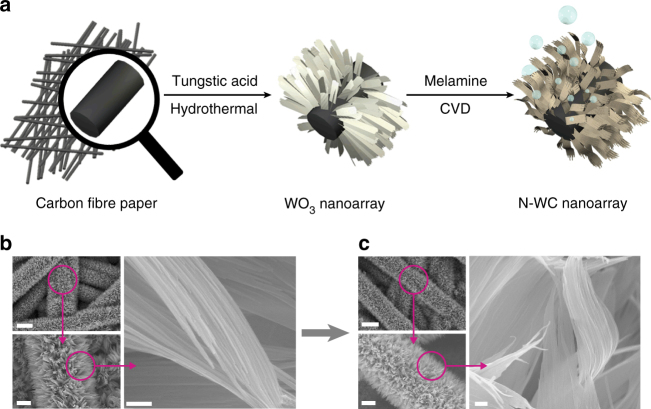
Fabrication of N-WC nanoarray electrodes for HER. **a** Schematic illustration: WO_3_ nanoarray on CFP were synthesized by a non-template, self-assembled hydrothermal reaction of tungstic acid; then the WO_3_ nanoarray was reduced, carbonized, and N-doped with melamine at high temperature to get N-WC nanoarray, which was used directly as high performance HER or OER catalytic electrode. **b** SEM of WO_3_ nanoarray. **c** SEM of N-WC nanoarray. Scale bar in **b** and **c**: left top 20 μm, left bottom 5 μm, right 200 nm

The N-doped WC nanoarray electrodes were prepared via a sequential two-step approach (Fig. 2a). In the first step, a controlled hydrothermal reaction of tungstic acid was used to grow WO_3_ nanoarray on carbon fiber paper (CFP). Subsequently, the WO_3_ nanoarray was subjected to reduction and simultaneously carbonization and N-doping at high temperature, using melamine as both C and N source. The chemical vapor deposition conditions were optimized to achieve high HER activity. The current densities for an applied potential of −200 mV vs. RHE were measured as indices. The optimal conditions that afford highest catalytic current density were found to be 850 °C for 3 h (Supplementary Fig. 3). The N-doped WC powder without nanoarray structure (N-WC), WC nanoarray without N-doping (WC nanoarray), and WC powder (WC) were also prepared at the optimized temperature and time to serve as control samples (see details in “Methods” section).

**Fig. 3:**
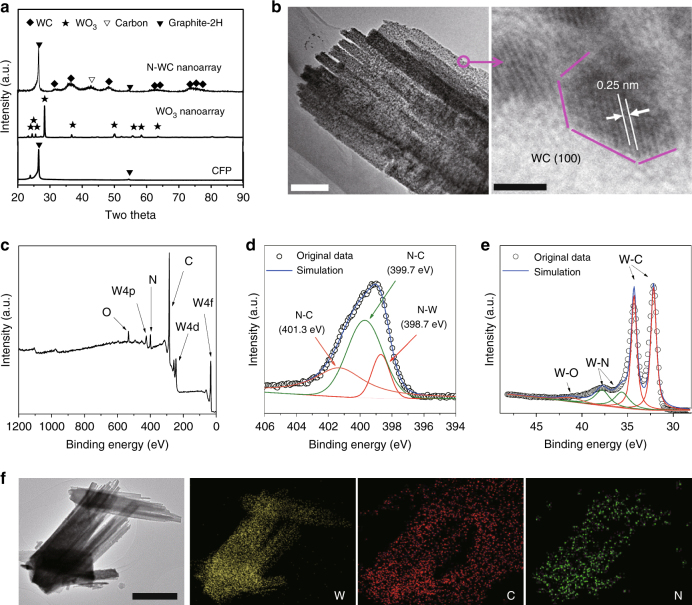
Characterization of N-WC nanoarray electrode. **a** XRD patterns of CFP and the synthesized WO3 nanoarray and N-WC nanoarray. **b** HRTEM of N-WC nanoarray. **c** XPS survey of N-WC nanoarray, and high-resolution XPS of **d** N 1s and **e** W 4f. **f** TEM-EDS element distribution of N-WC nanoarray. Scale bar in **b**: left 200 nm, right 2 nm. Scale bar in **f** 1 μm

Figure 2b shows the scanning electron microscopy (SEM) of WO_3_ grown on CFP, which exhibits a nanoarray structure composed of thin belts, with about 5 μm long, ~400 nm wide, and ~10 nm thick. The morphology of WO_3_ was well maintained after being converted to N-WC nanoarray (Fig. 2c) except for becoming 2–3 nm thinner. The perfect maintenance of nanoarray structure could be attributed to the thin-belt morphology that most oxygen atoms were near the surface, so oxygen atoms could be easily taken out and substituted by carbon or nitrogen atoms without damage. Figure 3a displays the X-ray diffraction (XRD) data of CFP, WO_3_ nanoarray, and N-WC nanoarray. In the XRD profile of CFP, the peaks at 26.4° and 42.5° are features of graphite (PDF#41–1487). The XRD pattern of WO_3_ nanoarray matches well with hexagonal WO_3_ (PDF#75–2187). After reduction and carbonization of WO_3_, hexagonal WC phase (PDF#51–0939) was successfully prepared as shown in the XRD pattern of N-WC nanoarray, in which the peak at 42.5° refers to extra tetragonal carbon (PDF#54–0501). The transmission electron microscopy (TEM) images of Fig. 3b show that each N-WC belt consists of parallel narrow belts arrayed side by side. On the surface, there are many nanoparticles with diameters of about 3 nm. The high-resolution TEM (HRTEM) image shows the lattice fringes of the nanoparticles. The 0.25 nm d-spacing corresponds to the (100) planes of hexagonal WC. WC (001) and WC (100) diffraction rings could be clearly seen in the SAED image (Supplementary Fig. 4). From SEM-energy dispersive spectrometer (EDS) mapping displayed in Supplementary Fig. 5, tungsten and carbon are the main components of N-WC nanoarray with only a very small amount of nitrogen. For comparison, Supplementary Fig. 6 shows XRD patterns of CFP, N-WC nanoarray, N-WC, and WC nanoarray. All of them behave as pure phases of WC (PDF#51–0939). The EDS of WC nanoarray in Supplementary Fig. 7 shows the absence of nitrogen.

X-ray photoelectron spectroscopy (XPS) data was performed to get more insights on the chemical environment of N-WC nanoarray. From the XPS data in Fig. 3c, the N element can be identified and the content at the surface of the sample is about 8.0 at.%. Figure 3d displays N 1s high-resolution XPS profile. The fitting peaks at 399.7 and 401.3 eV can be assigned to features of N–C bonds, and the peak at 398.7 eV has been reported to represent N-W bonding^44^. The presence of the N–W bond is also supported by the W 4f XPS in Fig. 3e. The peak at higher binding energy of 41.3 eV results from the inevitable surface oxidation of N-WC nanoarray upon exposure to air. Binding energy at 35.5 and 38 eV are supposed to be W–N bonds^45^, and the peaks centered at 32.5 and 34.6 eV are attributed to be W–C bonds^18,46^.

Figure 3f and Supplementary Table 1 show the TEM-EDS result and the corresponding element distribution of N-WC nanoarray. As shown in elemental mapping, N is uniformly distributed along the N-WC nanobelts. The TEM-EDS spectrum of N-WC nanoarray is displayed in Supplementary Fig. 8. According to the TEM-EDS result, the N content is about 6.6 at.%, which is close to the XPS survey result, indicating that the N element is uniformly doped inside the sample.

### Electrocatalytic hydrogen evolution

**Fig. 4:**
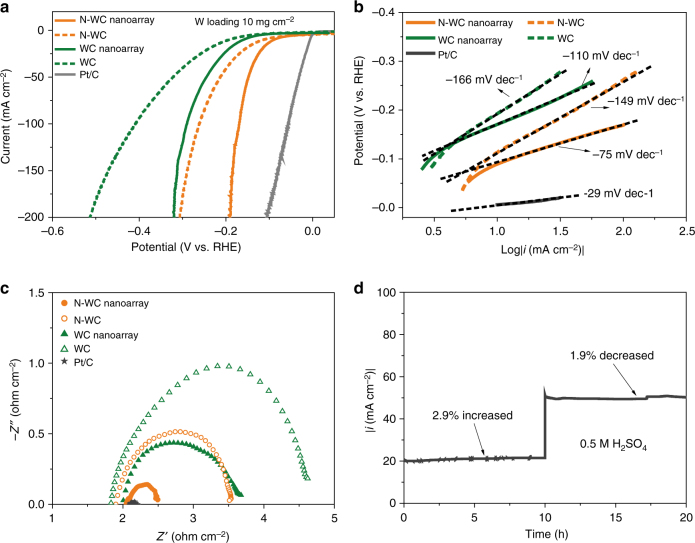
HER performance and comparison. **a** LSV of commercial Pt/C, WC, WC nanoarray, N-WC, and N-WC nanoarray in 0.5 M H_2_SO_4_ with iR correction at a scan rate of 1 mV/s. **b** Tafel plots calculated from **a**. **c** AC impedance of WC, WC nanoarray, N-WC, N-WC nanoarray, and commercial Pt/C catalysts. **d** Stability test of N-WC nanoarray at the overpotential of −0.144 V for 10 h, and then −0.156 V for another 10 h with iR correction

The electrocatalytic performance of N-WC nanoarray electrodes for HER was measured with three-electrode configuration in 0.5 M H_2_SO_4_. Figure 4a displays the linear scan voltammogram (LSV) curves for different electrocatalysts with iR correction. The metal loadings of the WC-based catalysts are all of 10 mg cm^−2^. The WC electrode exhibits catalytic performance with overpotential of −193 mV at −10 mA cm^−2^ and −513 mV at −200 mA cm^−2^, comparable to the WC catalyst usually reported^18,39^. After doping with nitrogen, the N-WC electrode shows much improved catalytic behavior, with −113 mV overpotential to drive catalytic current of −10 mA cm^−2^ and −310 mV overpotential for −200 mA cm^−2^. The N-doping effect confirmed the suggestion from DFT calculations showing that N in N-WC can make the W–H binding energy more suitable for HER kinetics.

Furthermore, the nanostructuring also brings favorable effects for catalytic performance. The WC nanoarray exhibits −200 mA cm^−2^ at −320 mV, showing improved catalytic performance over its WC counterpart (−513 mV). Most importantly, N-WC nanoarray shows the most impressive HER activity with small overpotentials of −89 mV at current density of −10 mA cm^−2^ and −190 mV overpotential at −200 mA cm^−2^ (Supplementary Table 2). Such a catalytic activity outperforms previously reported carbide-based electrodes (Supplementary Table 3). The performance of N-WC nanoarray is also outstanding among the best of non-precious metal-based HER electrocatalyst in acid (Supplementary Table 4). The electrochemical active surface areas (ECSA) data were derived from CV curves at non-Faraday area (see details in “Methods” section and Supplementary Fig. 9). Both the nanoarray samples show significant larger ECSA than the powder samples (Supplementary Fig. 10). After ECSA normalization, the superiority of N-WC samples is still remarkable, further demonstrating the improvement of intrinsic activity by N-doping (Supplementary Fig. 11). Also, the nanoarray samples show much higher activity than their powder counterparts, which implies that the nanoarray structure favors HER not only because of the enlarged surface area, but also due to the accelerated gas bubble departure.

As displayed in Fig. 4b, the Tafel slope of N-WC nanoarray for HER is −75 mV dec^−1^, which is the smallest among all the WC-based electrocatalysts, demonstrating a more favorable HER process of the N-WC nanoarray electrode. The fast HER reaction kinetics of N-WC is also reflected by a small charge transfer resistance of 0.5 ohms at the potential of −0.5 V vs. reversible hydrogen electrode (Fig. 4c).

The stability of N-WC nanoarray was tested at an overpotential of −0.144 V (~−20 mA cm^−2^) for the first 10 h, and then −0.156 V (~−50 mA cm^−2^) for another 10 h, as displayed in Fig. 4d. The current density increased by 2.9% at the first stage and then declined 1.9% at higher overpotential in the following 10 h of electrolysis. The excellent stability at −50 mA cm^−2^ has never been reported for tungsten carbide^18,35,36^. The increase of current density at −0.144 mV implies that the electrode is activated during HER first. Then, the decay of catalytic current appears in the next period. To verify this process, we further tested the stability of a fresh sample at about −60 mA cm^−2^. As depicted in Supplementary Fig. 12, the current density increased in the first 6 h and then decreased a little to an equilibrium state that the current remained unchanged throughout the rest 12 h. The current density decreased about 0.5% after the total 22 h test at −60 mA cm^−2^. To simulate the industrial production of H_2_ at large current density with interruptions, we performed interrupted stability test at −100 mA cm^−2^ (Supplementary Fig. 13). The current density decreased about 14% after 30 h electrolysis of water. The outstanding catalytic stability can be attributed to its high chemical stability and robustness of the nanoarray structure for bubble releasing. From the postmortem XPS analysis (Supplementary Fig. 14), excellent stability of N-WC nanoarray is further confirmed by the negligible structural differences before and after long-term electrolysis of water.

### Bubble behavior

**Fig. 5:**
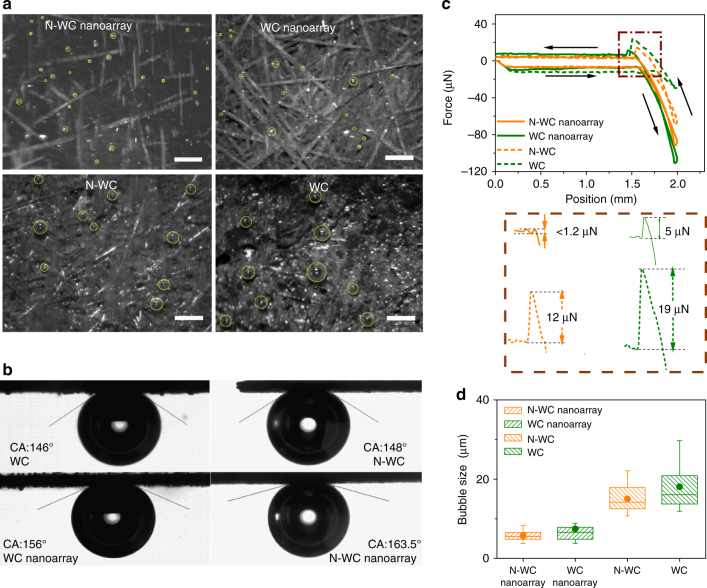
Bubble behavior study. **a** Snapshots of bubbles detaching from different electrodes at −200 mV vs. RHE. **b** Bubble contact angles, **c** bubble adhesion forces, and **d** bubble size distributions on different electrodes of WC, WC nanoarray, N-WC, and N-WC nanoarray. Scale bar in **a**: 100 μm

The bubble releasing behaviors during the electrocatalytic reaction at −200 mV vs. RHE were recorded with a high-speed camera system. It is clearly seen that WC and WC nanoarray exhibit less and slower H_2_ evolution than N-WC and N-WC nanoarray electrodes (Supplementary Movie 1 and snapshots in Fig. 5a). The observed significant difference shows that N-doping increases the intrinsic catalytic activity by modulating the electronic structure of WC as suggested by our computational predictions. Meanwhile, on the surfaces of WC and N-WC electrodes, we could see large areas of the light-reflecting gas films which result from H_2_ gas strongly adhered on the electrode surface. Once the active sites are isolated from the aqueous electrolyte by adhered bubbles or gas films, the electrocatalytic reaction slows down. In contrast, there was no obvious gas film or large bubbles adhered on the surface of WC nanoarray or N-WC nanoarray, thus the catalytic HER continues without interruption. Furthermore, the bubbles on N-WC nanoarray are much smaller and leave much faster than bubbles on the other electrodes.

The significant differences in the bubble behaviors suggest a weaker adhesion of bubbles at the nanoarray electrode than on the flat electrode, which is quantitatively described by the contact angle (CA) and adhesion force (AF). As shown in Fig. 5b and c, non-nanostructured electrodes show CA = 146° and AF = 19 μN for WC, while CA = 148° and AF = 12 μN for N-WC. In contrast, the two nanoarray electrodes show significant increases in CA (156° for WC nanoarray and 163° for N-WC nanoarray) and decreases in AF (5 μN for WC nanoarray, and almost undetectable for N-WC nanoarray). These data support the fact that surface roughening can improve the catalytic HER activity by weakening the interactions of the bubbles with the electrode surface, thus favoring rapid release of H_2_ bubbles. Furthermore, the N-doped WC electrodes show a slightly larger CA and weaker AF than their non-N-doped WC counterparts of the same structure, showing further evidence that N-doping weakens the force between H_2_ bubbles and the electrode surface.

The size distributions of bubbles detached from the electrodes are quantified statistically in Fig. 5d. The average bubble sizes on WC nanoarray and N-WC nanoarray are not distinctly different, and similar observation applies to WC and N-WC electrodes. However, when the nanoarray electrodes are compared to their non-structured counterparts, the advantages are clear—smaller bubble size along with larger CA and weaker AF. Accordingly, the WC nanoarray exhibits much better catalytic activity than WC, while N-WC nanoarray outperforms N-WC. Therefore, the accelerating effect of nanoarray structuring toward HER is further confirmed.

### Electrocatalytic oxygen evolution

**Fig. 6:**
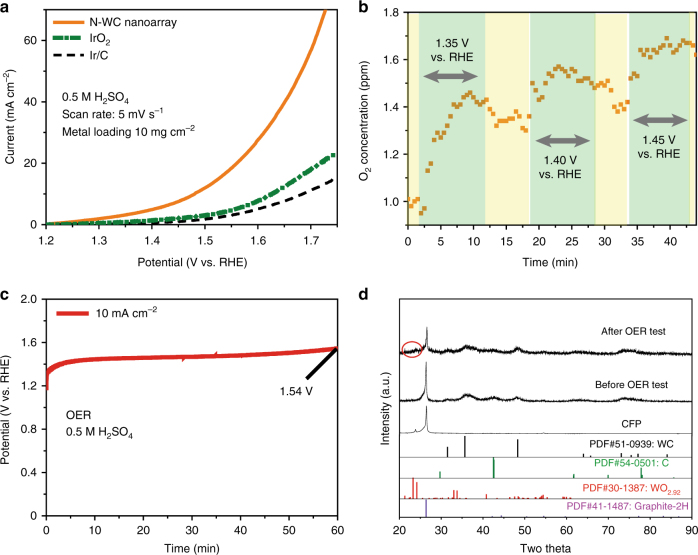
OER performance and comparison. **a** OER polarization curves tested in 0.5 M H_2_SO_4_ of N-WC nanoarray, IrO_2_, and 20 wt.% Ir/C at a scan rate of 5 mV/s with iR correction. **b** O_2_ concentration in 0.5 M H_2_SO_4_ when different potentials are intermittently applied to N-WC nanoarray electrode. **c** Stability test of N-WC nanoarray for OER at 10 mA cm^−2^. **d** XRD patterns of N-WC nanoarray before and after OER test

In 0.5 M H_2_SO_4_, the OER performance of N-WC nanoarray was measured with a Pt foil as the counter electrode, in comparison with commercial 20 wt.% Ir/C and IrO_2_ catalyst. The metal loadings of the three electrodes are all of 10 mg cm^−2^. As shown in Fig. 6a, OER starts at about 1.35 V on N-WC nanoarray electrode, and about 1.5 V on the commercial Ir/C and IrO_2_ catalyst. The current density on N-WC nanoarray increases very fast with potential. At about 1.7 V vs. RHE, the current density reaches up to 60 mA cm^−2^. The O_2_ concentration in the electrolyte was detected by a dissolved oxygen meter (DOM) during the electrochemical test when potentials of 1.35, 1.4, and 1.45 V were intermittently applied to the N-WC nanoarray. In Fig. 6b, the O_2_ concentration rises up to 1.5, 1.6, and 1.7 ppm, respectively, when 1.35, 1.4, and 1.45 V voltage is applied to the electrode and falls every time when the voltage is off. The synchronized variation between voltage and O_2_ concentration indicates that OER really occurs at about 1.35 V vs. RHE. Although the OER activity of N-WC nanoarray is impressive, the stability of N-WC nanoarray for OER in acid still needs to be improved, as shown in Fig. 6c. The potential increases from 1.35 to 1.54 V vs. RHE after 1 h of continuous water splitting at 10 mA cm^−2^. To acquire the reason for the instability of N-WC nanoarray, we recorded the XRD patterns of N-WC nanoarray before and after OER test, showing that a small amount of tungsten oxides formed on N-WC nanoarray during OER test (Fig. 6d). Although N-WC nanoarray is not stable enough for practical application for now, the N-WC-based anode would represent an important research avenue for non-precious OER catalyst in acid.

### Electrocatalytic water splitting

**Fig. 7:**
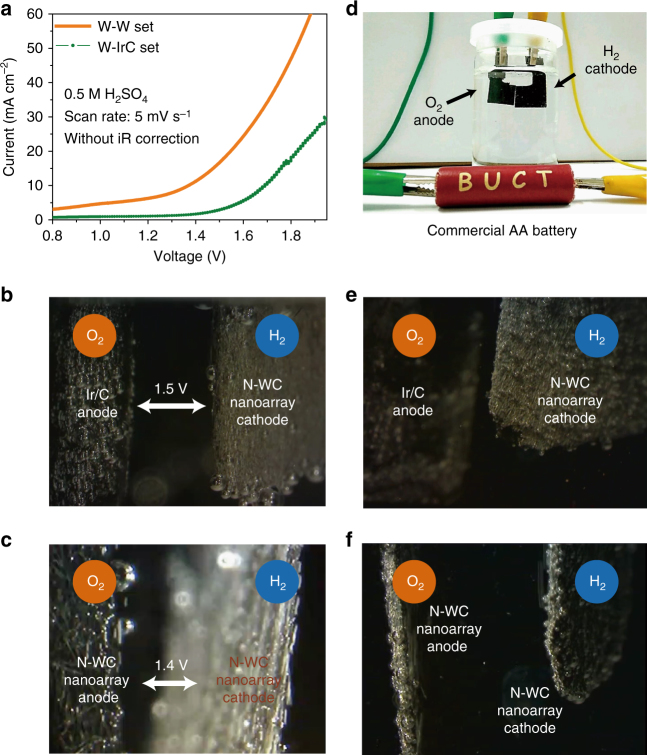
Overall water splitting. **a** The Volt-ampere curves of overall water splitting with N-WC nanoarray as the cathode, while using Ir/C (noted as W–IrC set) or N-WC nanoarray (noted as W–W set) as the anode. **b** The video snapshot of water electrolysis by W–IrC set with voltage at 1.5 V. **c** The video snapshot of water electrolysis by W–W set with voltage at 1.4 V. **d** Setup for overall water splitting powered by a commercial AA battery, and the corresponding snapshots of gas bubbling with water electrolysis of W–IrC set (**e**) or W–W set (**f**)

With N-WC nanoarray as the cathode, either N-WC nanoarray (electrolysis cell noted as W–W set) or commercial Ir/C (loading of 5 mg cm^−2^) electrode (electrolysis cell noted as W–IrC set) as the anode, the overall water splitting performance was measured. At room temperature (20 °C), the experiment was carried out in 0.5 M H_2_SO_4_, and the corresponding Volt-ampere curves are displayed in Fig. 7a. In terms of the W–IrC set, the overall water splitting reaction begins at about 1.5 V. We conducted a chronoamperometry test at 1.5 V without iR compensation and recorded the video of the electrodes (Supplementary Movie 2). From the snapshot displayed in Fig. 7b, bubbles can be seen clearly on both the anode and the cathode, consistent with water splitting starting at about 1.5 V. The N-WC nanoarray thus show promise for practical applications as a HER cathode. Surprisingly, the overall water splitting of the W–W set begins at about 1.4 V. Before 1.7 V vs. RHE, the current density reaches up to 30 mA cm^−2^. These results are significant since such an excellent performance for catalytic water electrolysis in acid has never been reported with non-noble metal catalysts. The chronoamperometry test of W–W set at 1.4 V without iR compensation was conducted. A recorded video of the electrodes (Supplementary Movie 3) shows bubbling vigorously on the electrodes (the snapshot in Fig. 7c), confirming high-efficient catalytic water splitting with N-WC nanoarrays as the bifunctional catalytic electrode for both the anode and the cathode. Furthermore, we tested water splitting performance of the W–IrC set and the W–W set powered by a commercial AA battery, as shown in Fig. 7d and corresponding videos (Supplementary Movies 4 and 5). Bubbles evolve from the electrodes of W–IrC set (Supplementary Movie 6, Fig. 7e), and more violently on the electrodes of W–W set (Supplementary Movie 7, Fig. 7f).

## Discussion

In summary, we have designed, constructed, and demonstrated a superaerophobic N-WC nanoarray catalyst for water splitting into H_2_ and O_2_ via controlled synthesis of WO_3_ nanoarray, followed by simultaneous reduction, carbonization, and N-doping with melamine at high temperature. To make a clear cut, N-doping plays the most important role in elevating the HER activity of N-WC nanoarray by improving the intrinsic activity at atomic level. At the same time, nanostructuring also boots the HER activity and stability by offering large effective surface area and promoting the release of gas evolution in nano-scale design. As a result, the N-WC nanoarray catalyst achieves catalytic current densities of −10 mA cm^−2^ at overpotentials of −89 mV and −200 mA cm^−2^ at −190 mV, featuring outstanding HER catalytic activity and working stability for long-term water electrolysis. The observed HER catalytic performance of our N-WC nanoarray is among the best HER catalysts based on non-noble metals in acid. More important, the overall water splitting reaction starts at about 1.4 V, with N-WC nanoarray working as both cathode and anode electrodes, showing low overpotential and high activity for the OER in acid.

## Methods

### Materials

Tungstic acid was purchased from Aladdin Industrial Corporation. Anhydrous sodium sulfate, hydrogen peroxide, and hydrochloric acid were purchased from Beijing Chemical Works. 5 wt.% Nafion D-521 dispersion, 20 wt.% Pt/C, IrO_2_, nano graphite, camphora, 20 wt.% Ir/C, and CFP were purchased from Alfa Aesar. Melamine was from Tianjin Guang Fu Fine Chemical Research Institute. All the solutions in this work were prepared with deionized water.

### Synthesis of WO_3_ nanoarrays

WO_3_ nanoarrays on CFP were synthesized by the hydrothermal method. With magnetic stirring, tungstic acid powder (0.625 g) was dissolved in 20 ml 12 wt.% hydrogen peroxide solution by heating at 95 °C for 3 h. Then, the solution was transferred into a Teflon reaction kettle with 0.2 g anhydrous sodium sulfate, 115 μl hydrochloric acid, and a piece of 2 cm × 2 cm CFP added in. WO_3_ nanoarrays were obtained after being heated for 12 h at 180 °C. WO_3_ powder without nanoarray structure was also synthesized by the same method without CFP.

### Reduction, carbonization, and N-doping

WO_3_ nanoarrays on a combustion boat that containing melamine was heated at various temperatures (*T*) and time (*t*) periods, with Ar gas flowing in a tube furnace. To optimize the catalytic performance, we tried *T* = 650, 750, 850, and 950 °C, and *t* = 0.5, 1, 3, and 6 h. The current densities (without iR correction) at overpotential of −200 mV were measured as indices. The optimal condition to afford highest catalytic current density was found to be 850 °C for 3 h (Supplementary Fig. 3). The N-WC powder without nanoarray structure was using WO_3_ powder as the precursor, carbonized and N-doped at the same condition with the best performed N-WC nanoarrays. WC nanoarrays and WC was made at the same temperature and time with N-WC nanoarrays separately, except using Camphora as the carbon source.

### Materials characterization

The morphologies were examined by JEOL JEM 2100 operating at 200 kV and Zeiss SUPRA-55 operating at 20 kV. XPS were carried out by using Thermo Electron ESCALAB-250. XRD patterns were acquired by Shimadzu XRD-6000 at 10(°)/min.

### Electrochemical measurements

Electrochemical measurements were performed in 0.5 M H_2_SO_4_ solution by a CH Instrument (CHI660E) with Ag/AgCl reference and graphite rod counter electrode during HER test. The Ag/AgCl electrode was corrected with a reversible hydrogen electrode and rinsed with deionized water before use. WC nanoarrays (20 mg cm^−2^) and N-WC nanoarrays (16 mg cm^−2^) were used as electrodes directly. Powder sample-based electrodes were made by the following steps: 16 mg of N-WC powder (or 20 mg WC powder) and 10 μl Nafion dispersion were mixed with 1 ml isopropanol to form catalyst ink which was then spread on a piece of CFP drop by drop under an infrared lamp. The metal loadings of the WC-based catalysts are all of 10 mg cm^−2^. Pt/C electrode was made by the same procedure except that 2 μl Nafion dispersion and 0.4 mg 20 wt.% Pt/C were mixed with 0.5 ml isopropanol to form catalyst ink. The loading of Pt/C electrode was 0.4 mg cm^−2^. Following the same procedure, Ir/C and IrO_2_ electrodes were made. 50 mg 20 wt.% Ir/C and 100 μl Nafion dispersion were mixed with 5 ml isopropanol to form Ir/C catalyst ink. IrO_2_ catalyst ink was made by 11.7 mg IrO_2_ and 20 μl Nafion dispersion mixed with 5 ml isopropanol. The metal loadings of Ir/C and IrO_2_ electrode were 10 mg cm^−2^. LSV for HER was performed at 1 mV s^−1^. LSV for OER was performed at 5 mV s^−1^. ECSA data were derived from CV curves of WC-based catalysts at non-Faraday area with different scan rates. At −0.5 V vs. RHE, EIS was measured from 100 kHz to 0.005 Hz with amplitude of 10 mV. Loadings of all catalysts are listed in Supplementary Table 5.

### Bubble behavior study

At room temperature, CA of hydrogen bubbles on different electrodes in 0.5 M H_2_SO_4_ were measured in ambient air by the captive-bubble method^47^ and the bubble size was controlled at 2 μl. AF between hydrogen bubbles and electrodes in 0.5 M H_2_SO_4_ was tested by Dataphysics DCAT21, Germany 20. The video for hydrogen evolution process was recorded by Microscope (SZ-CTC, OLYMPUS) with X-Motion, AOS Technologies.

### O_2_ concentration test

O_2_ concentration in 0.5 M H_2_SO_4_ was tested by DOM (TP351 Lab Dissolved Oxygen Meter). DOM was turn on for 8 h to reach balance in air before use. 0.5 M H_2_SO_4_ was purged with Ar gas for 1 h before test.

### Statistics

In terms of Fig. 5d, 50 bubbles detaching from the surface of each electrode were randomly recorded and used directly in the box plots.

### DFT calculations

All periodic boundary calculations were performed by Vienna ab initio simulation package^48–51^. To describe the electron–ion interactions, projector augmented plane wave method^52,53^ and the PBE exchange-correlation functional^54^ were used. For the plane wave basis set in all calculations, a cutoff of 450 eV was chosen. For the optimization of bulk Pt and WC, a 7 × 7 × 7 Monckhorst-Pack type k-point grid was chosen. For the calculation of Pt (111) and WC (001) slabs, a 5 × 5 × 1 Monckhorst-Pack type k-point grid was chosen. The second-order Methfessel-Paxton was used for Brillouin-zone integration^29^ and the *σ-*value was chosen to be 0.1 eV. For structure optimization, the force convergence criterion of 0.01 eV Å^−1^ was used. The energy convergence criterion was set to be 10^−4^ eV per unit cell. To model the Pt (111) surface, five layers of Pt atoms and four Pt atom per layers were used. The bottom three layers were fixed during optimization. Four layers of W and four layers of C atoms and four atoms per layer were used to model the WC (001) surface. To model the N-WC (001) surface, we replaced half of the subsurface C atoms with N atoms, which corresponding to an atomic percentage of N atoms of 6.25%, close to the experimental atomic percentage of 6.61%. The bottom two layers of W and C atoms were fixed during geometry optimization for WC (001) and N-WC (001) surfaces. A vacuum slab of about 15 Å was inserted between the surface slab. A supercell of 15 Å × 15 Å × 15 Å and a 1 × 1 × 1 Monkhorst-Pack k-point mesh was employed for the calculation of H_2_. Only one hydrogen atom was included in the calculation of the hydrogen absorbed surfaces, corresponding to a surface coverage of ¼. The HBE was calculated as HBE = *E*_total_–0.5*E*_H2_–*E*_surf_. *E*_total_ is the absorbed system’s total energy, *E*_surf_ is the optimized bare surface’s energy, and *E*_ads_ is the energy of the adsorbate (H_2_) in vacuum.

### Data availability

Upon reasonable request, more data are available from the corresponding author.

## Electronic supplementary material

Supplementary Information

Description of Additional Supplementary Files

Supplementary Movie 1

Supplementary Movie 2

Supplementary Movie 3

Supplementary Movie 4

Supplementary Movie 5

Supplementary Movie 6

Supplementary Movie 7
