# Supplementary material for: Nitrogen-doped tungsten carbide nanoarray as an efficient bifunctional electrocatalyst for water splitting in acid
Source: Nat Commun. 2018 Mar 2;9:924. doi: 10.1038/s41467-018-03429-z (PMC5834627; doi:10.1038/s41467-018-03429-z)
Supplement: Supplementary file 1 — Supplementary Information [file 41467_2018_3429_MOESM1_ESM.pdf]

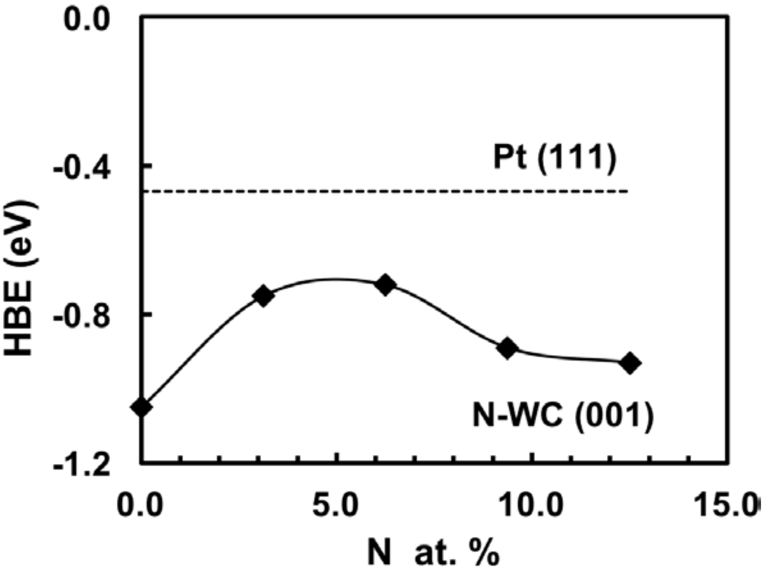

Supplementary Figure 1 | Correlation of hydrogen binding energy (HBE) with different N doping amount on N-WC (001). The 6.25 % N doping amount shows the most appropriate HBEs in N-WC.

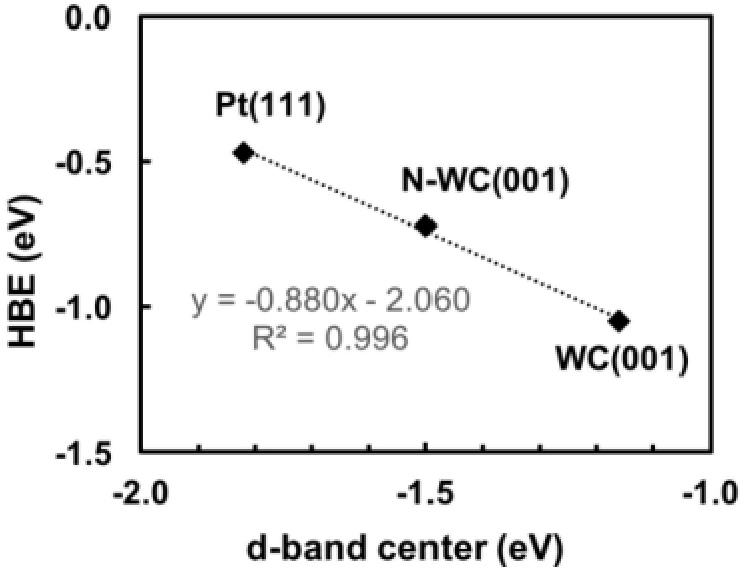

Supplementary Figure 2 | Correlation of hydrogen binding energy (HBE) at 1/4 coverage with calculated d-band centers on Pt (111), N-WC (001), and WC (001). The coefficient of determination R^2 indicates strong correlation between hydrogen binding energy and d-band centers.

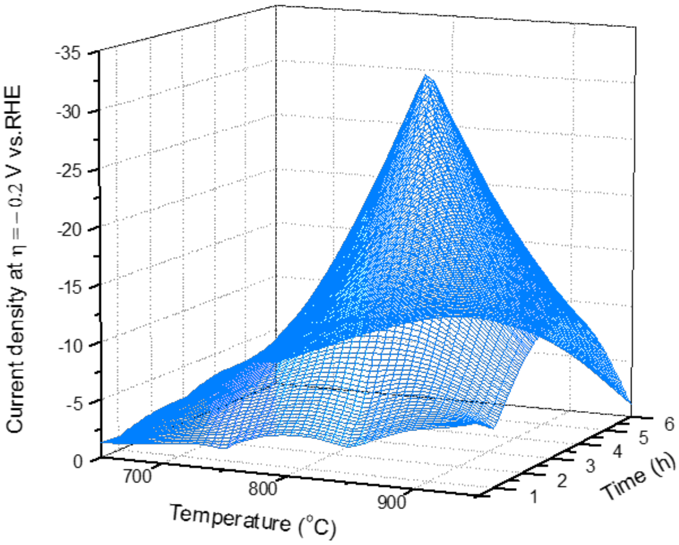

Supplementary Figure 3 | Optimization of CVD synthesis conditions for N-WC nanoarray. To optimize the catalytic performance, we tried T= 650, 750, 850 and 950 °C, and t = 0.5, 1, 3 and 6 h. Current densities (without iR correction) at the overpotential of -200 mV for HER were read from their linear scan voltammogram (LSV) tests. The result is displayed in the above figure, which shows that the sample carbonized and N-doped at the condition of T=850 °C and t=3 h has the highest current density, i.e., the best performance.

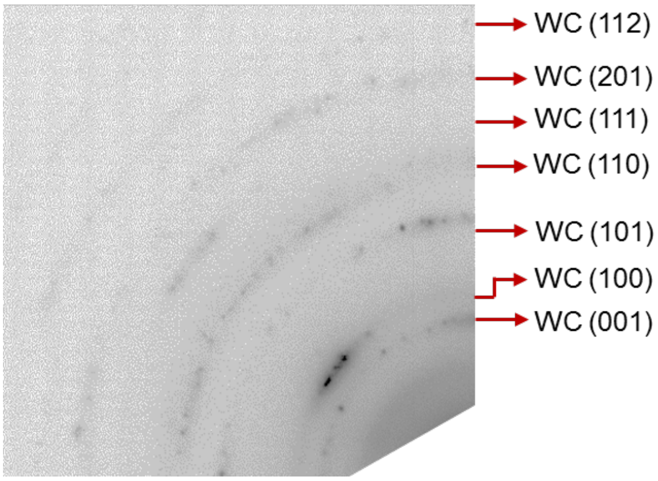

Supplementary Figure 4 | TEM SAED graph of N-WC nanoarray.

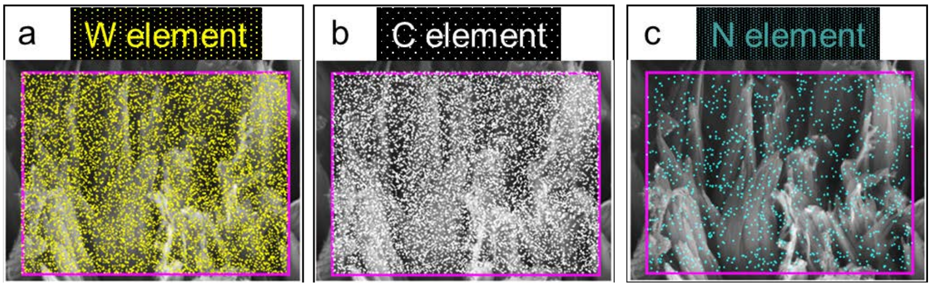

Supplementary Figure 5 | EDS mapping of N-WC nanoarray. The elemental distribution of W **a**, C **b** and N **c** of N-WC. Tungsten and carbon are the main content of N-WC nanoarray, and only a very small amount of nitrogen exist.

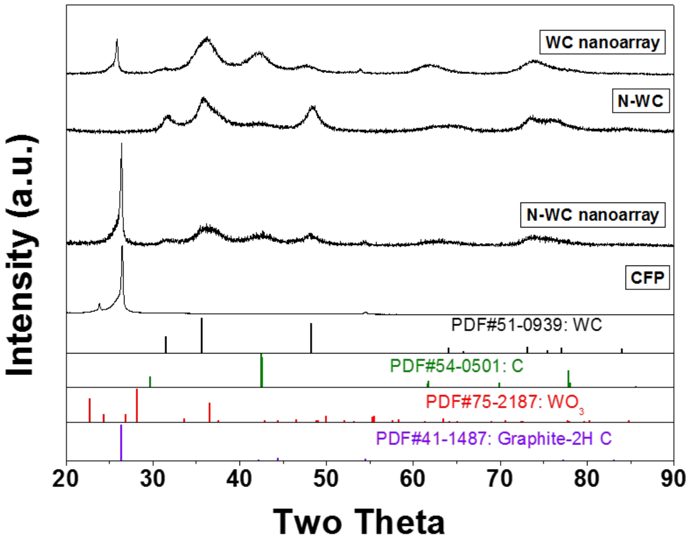

Supplementary Figure 6 | XRD patterns of CFP, N-WC nanoarray, N-WC, and WC nanoarray. The XRD patterns of the samples are almost the same with WC phase (PDF#51-0939).

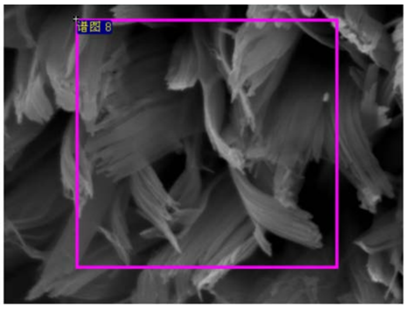

element	Weight ratio	Atomic ratio
**C K**	22.69	80.40
**N K**	0.00	0.00
**O K**	0.70	1.86
**W M**	76.61	17.74
**total**	100.00	100.00

Supplementary Figure 7| SEM-EDS results of WC nanoarray. The morphology of WC nanoarray is almost the same with N-WC nanoarray in SEM, while N is absent in WC nanoarray from the quantity analysis.

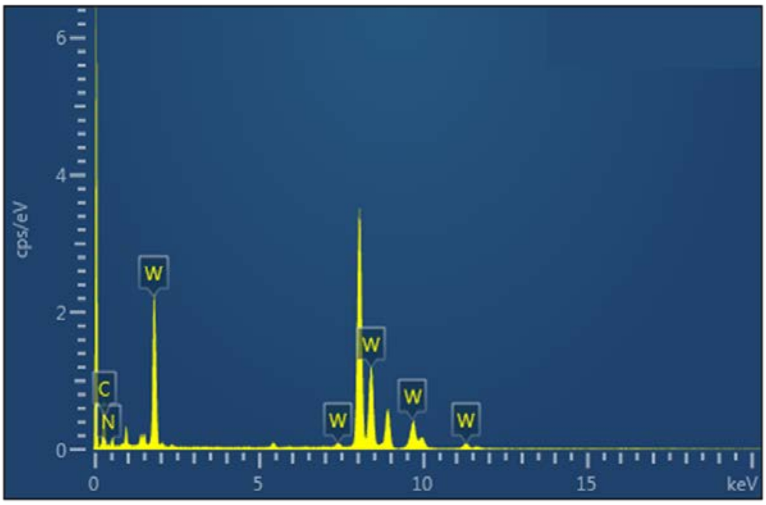

Supplementary Figure 8 | TEM-EDS spectrum of N-WC nanoarray. W and C are the main components while only 6.6 at. % of N exists in N-WC nanoarray sample.

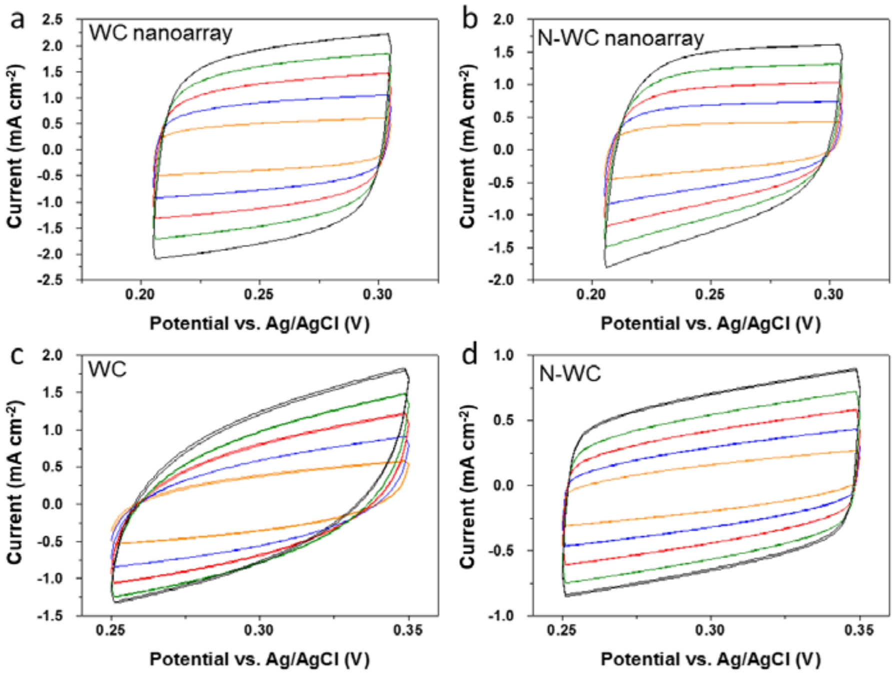

Supplementary Figure 9 | CV curves of the WC based catalysts at non-Faraday area. The CV curves were tested in 0.5 M H_2SO_4 at potential range without chemical reaction under scan rates of 5, 10, 15, 20 and 25 mV s^{-1}. **a**, **b**, **c** and **d** are CV curves of WC nanoarray, N-WC nanoarray, WC and N-WC, respectively.

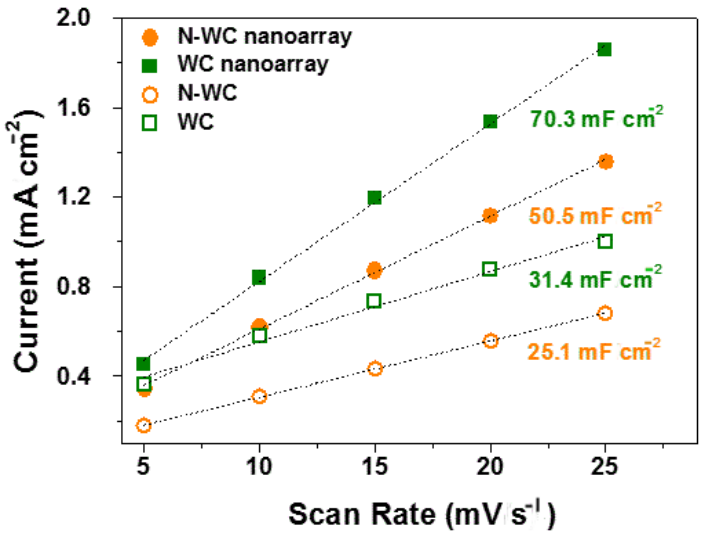

Supplementary Figure 10 | ECSA of the WC based catalysts. Both of the two nanoarray-structure samples display larger ECSA than their powder counterparts. The enlarged ECSA favors HER with more active sites.

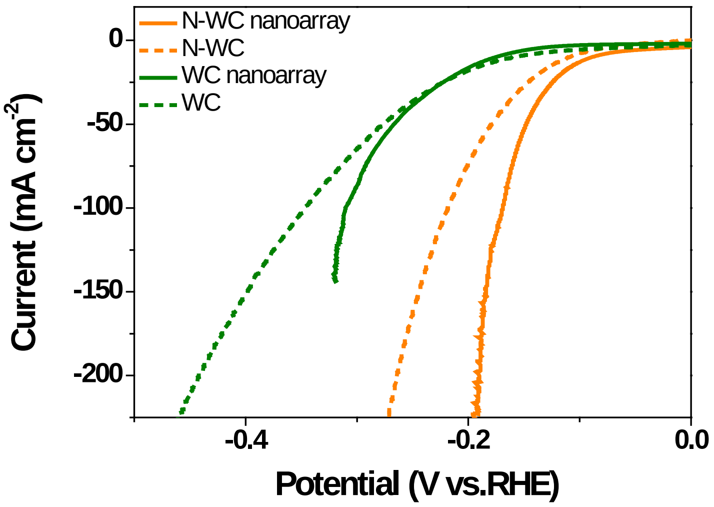

Supplementary Figure 11 | ECSA-normalized HER polarization curves of the WC based catalysts. After ECSA normalization, the superiority of N-WC samples is still remarkable, further demonstrating the improvement of intrinsic activity by N doping. Also, the nanoarray samples show much higher activity than their powder counterparts, which implies that the nanoarray structure favors HER not only because of the enlarged surface area, but also due to the accelerated gas bubble departure.

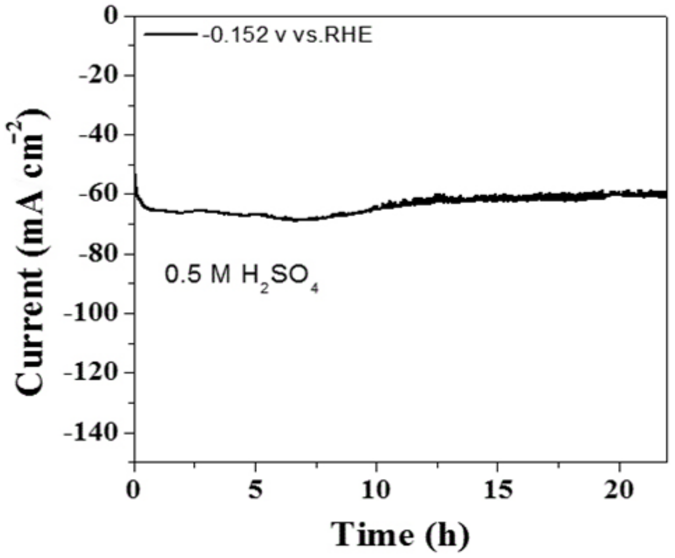

Supplementary Figure 12|Stability test at about -60 A cm^{-2}. The stability of a fresh sample at about -60 mA cm^{-2} was tested. The current density increased in the first 6 h and then decreased a little to an equilibrium state that the current remained unchanged throughout the rest 12 h. The current density decreased about 0.5% after the total 22h test at about -60 mA cm^{-2}.

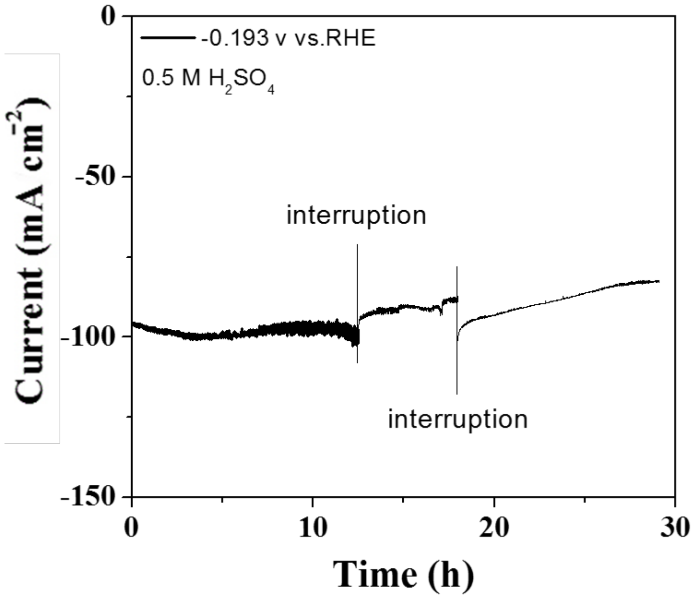

Supplementary Figure 13|Interrupted stability test at about -100 mA cm^{-2}. To simulate industrial production of H_2 at large current density with interruptions, we performed interrupted stability test at about -100 mA cm^{-2}. The current density decreased about 14% after about 30 h test.

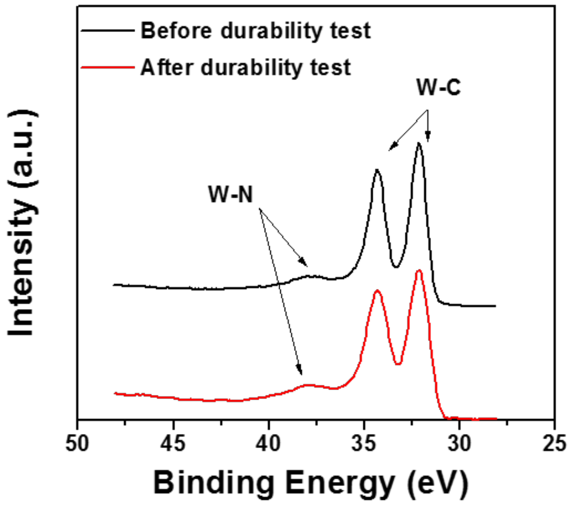

Supplementary Figure 14 | High resolution XPS profiles of W4f for N-WC nanoarray before and after stability test. The height and position of peaks for W-C and W-N are almost the same before and after stability test which implies that the N-WC nanoarray catalyst possesses good stability.

Supplementary Table 1| TEM-EDS result of N-WC nanoarray.

Element	wt%	at%
W	89.61	36.43
C	9.15	56.96
N	1.24	6.61
Total	100.00	100.00

Supplementary Table 2 | Performance of the four WC based HER catalysts discussed in this article. The loadings, electrochemical specific surface area (ECSA), and overpotentials at -10, -50, and -200 mA cm^{-2} are compared in the table below.

Sample	Loading (mg)	ECSA (mF cm^{-2})	η@-200mA cm^{-2} (mV)	η@-50mA cm^{-2} (mV)	η@-10mA cm^{-2} (mV)
N-WC nanoarray	16	50.5	-190	-148	-89
N-WC	16	25.1	-306	-213	-114
WC nanoarray	20	70.3	-319	-245	-160
WC	20	31.4	-513	-322	-193

Supplementary Table 3 | Performance of metal carbide based electrodes for HER in acid. Catalysts with the working **c**urrent density as high as -200 mA cm^{-2} were rarely reported. Although Ref. 1 tested MoC to the current density of -200 mA cm^{-2}, the overpotential is too large during the whole current density range. The N-WC nanoarray electrode is therefore outstanding among the metal carbide based HER catalysts as activity is concerned in both low and high current densities. (Overpotential data were read from the LSV curves in each corresponding reference.)

Materials	η@-200mA cm^{-2} (mV)	η@-50mA cm^{-2} (mV)	η@-10mA cm^{-2} (mV)	Reference No.
**N-WC nanoarray**	**-190**	**-148**	**-89**	**This Work**
**MoC**	**-234**	**-177**	**-133**	1
**WC**	N/A	**-112**	**-53**	2
**MoCx**	N/A	**-126**	**-73**	3
**W_2C+WN**	N/A	N/A	**-120**	4
**W_2C**	N/A	N/A	**-120**	5
**Mo_2C**	N/A	N/A	**-220**	6
**Fe-WCN**	N/A	N/A	**-250**	7

Supplementary Table 4 | Performance of important non-precious metal based electrocatalysts for HER in acid. Catalysts with the working current density to -200 mA cm^{-2} were only found in four references, among which only MoP_2 displayed higher activity than N-WC nanoarray yet only with long term stability test at current density of -20 mA cm^{-2}. Therefore, both the activity and stability displayed by N-WC nanoarray are excellent among the non-precious metal based electro-catalysts for HER in acid. (Overpotential data were read from the LSV curves in each corresponding reference.)

Materials	η@-200mA cm^{-2} (mV)	η@-50mA cm^{-2} (mV)	η@-10mA cm^{-2} (mV)	Reference No.
N-WC nanoarray	-190	-148	-89	This Work
MoP_2	-140	-100	-63	8
CoSe	-190	-168	-138	9
MoC	-234	-177	-133	1
MoS	- 265	-226	-183	10
WC	N/A	-112	-53	2
MoP|S	N/A	-100	-63	11
MoCx	N/A	-126	-73	3
CoP	N/A	N/A	-83	12
CoSe	N/A	-171	-113	13
W_2C+WN	N/A	N/A	-120	4
W_2C	N/A	N/A	-120	5
WS_2	N/A	N/A	-160	14
TiN	N/A	N/A	-170	15
$CoTe_2$	N/A	-213	-173	16
Co_9S_8	N/A	-290	-180	17
MoS_2	N/A	N/A	-219	18
Mo_2C	N/A	N/A	-220	6
Fe-WCN	N/A	N/A	-250	7
Co_8S_8	N/A	-700	-280	19
FeCo@NCNTs-NH	N/A	N/A	-280	20

Supplementary Table 5| Mass loadings and metal loadings of catalysts for HER and OER.

	N-WC nanoarray	N-WC	WC nanoarray	WC	Pt/C (20 wt. %)	Ir/C (20 wt. %)	IrO_2
**Catalyst loading**	16 mg/cm^2	16 mg/cm^2	20 mg/cm^2	20 mg/cm^2	0.4 mg/cm^2	50 mg/cm^2	11.7 mg/cm^2
**Metal loading**	*10 mg/cm^2	10 mg/cm^2	*10 mg/cm^2	10 mg/cm^2	0.08 mg/cm^2	10 mg/cm^2	10 mg/cm^2

*The metal loadings of N-WC nanoarray and WC nanoarray were calculated from the mass of the precursor WO_3 nanoarray which was 13.1 mg/cm^2.

Supplementary References.

1. Shi Z*, et al.* Porous nanoMoC@graphite shell derived from a MOFs-directed strategy: an efficient electrocatalyst for the hydrogen evolution reaction. *J. Mater. Chem. A* **4**, 6006—6013 (2016).
2. Xu Y-T*, et al.* Cage-Confinement Pyrolysis Route to Ultrasmall Tungsten Carbide Nanoparticles for Efficient Electrocatalytic Hydrogen Evolution. *J. Am. Chem. Soc.* **139**, 5285—5288 (2017).
3. Yang X*, et al.* N-Doped graphene-coated molybdenum carbide nanoparticles as highly efficient electrocatalysts for the hydrogen evolution reaction. *J. Mater. Chem. A* **4**, 3947—3954 (2016).
4. Chen W-F*, et al.* Tungsten Carbide-Nitride on Graphene Nanoplatelets as a Durable Hydrogen Evolution Electrocatalyst. *ChemSusChem* **7**, 2414—2418 (2014).
5. Gong Q*, et al.* Ultrasmall and phase-pure W_2C nanoparticles for efficient electrocatalytic and photoelectrochemical hydrogen evolution. *Nat. Commun.* **7**, 13216—13223 (2016).
6. Vrubel H, Hu X. Molybdenum Boride and Carbide Catalyze Hydrogen Evolution in both Acidic and Basic Solutions. *Angew. Chem. Int. Ed.* **51**, 12703—12706 (2012).
7. Zhao Y, Kamiya K, Hashimoto K, Nakanishi S. Hydrogen Evolution by Tungsten Carbonitride Nanoelectrocatalysts Synthesized by the Formation of a Tungsten Acid/Polymer Hybrid In Situ. *Angew. Chem. Int. Ed.* **52**, 13638—13641 (2013).
8. Zhu W, Tang C, Liu D, Wang J, Asiri AM, Sun X. A self-standing nanoporous MoP_2 nanosheet array: an advanced pH-universal catalytic electrode for the hydrogen evolution reaction. *J. Mater. Chem. A* **4**, 7169—7173 (2016).
9. Kong D, Wang H, Lu Z, Cui Y. $CoSe_2$ Nanoparticles Grown on Carbon Fiber Paper: An Efficient and Stable Electrocatalyst for Hydrogen Evolution Reaction. *J. Am. Chem. Soc.* **136**, 4897—4900 (2014).
10. Kibsgaard J, Jaramillo TF, Besenbacher F. Building an appropriate active-site motif into a hydrogen-evolution catalyst with thiomolybdate $[Mo_3S_{13}]^{2-}$ clusters. *Nat.Chem.* **6**, 248—253 (2014).
11. Kibsgaard J, Jaramillo TF. Molybdenum Phosphosulfide: An Active, Acid-Stable, Earth-Abundant Catalyst for the Hydrogen Evolution Reaction. *Angew. Chem. Int. Ed.* **53**, 14433—14437 (2014).
12. Saadi FH, Carim AI, Verlage E, Hemminger JC, Lewis NS, Soriaga MP. CoP as an Acid-Stable Active Electrocatalyst for the Hydrogen-Evolution Reaction: Electrochemical Synthesis, Interfacial Characterization and Performance Evaluation. *J. Phys. Chem. C* **118**, 29294—29300 (2014).
13. Lee C-P*, et al.* Beaded stream-like $CoSe_2$ nanoneedle array for efficient hydrogen evolution electrocatalysis. *J. Mater. Chem. A* **4**, 4553—4561 (2016).
14. Cheng L*, et al.* Ultrathin WS_2 Nanoflakes as a High-Performance Electrocatalyst for the Hydrogen Evolution Reaction. *Angew. Chem. Int. Ed.* **53**, 7860—7863 (2014).
15. Han Y, Yue X, Jin Y, Huang X, Shen PK. Hydrogen evolution reaction in acidic media on single-crystalline titanium nitride nanowires as an efficient non-noble metal electrocatalyst. *J. Mater. Chem. A* **4**, 3673—3677 (2016).
16. Wang K*, et al.* Morphology-Controllable Synthesis of Cobalt Telluride Branched Nanostructures on Carbon Fiber Paper as Electrocatalysts for Hydrogen Evolution Reaction. *ACS Appl. Mater. Interfaces* **8**, 2910—2916 (2016).
17. Feng L-L*, et al.* Metallic Co_9S_8 nanosheets grown on carbon cloth as efficient binder-free electrocatalysts for the hydrogen evolution reaction in neutral media. *J. Mater. Chem. A* **4**, 6860—6867 (2016).
18. Deng H*, et al.* Laser induced MoS_2/carbon hybrids for hydrogen evolution reaction catalysts. *J.*

Mater. Chem. A **4**, 6824—6830 (2016).

19. Feng L-L, *et al.* Carbon-Armored Co_9S_8 Nanoparticles as All-pH Efficient and Durable H_2-Evolving Electrocatalysts. *ACS Appl. Mater. Interfaces* **7**, 980—988 (2015).
20. Deng J, Ren P, Deng D, Yu L, Yang F, Bao X. Highly active and durable non-precious-metal catalysts encapsulated in carbon nanotubes for hydrogen evolution reaction. *Energy Environ. Sci.* **7**, 1919—1923 (2014).
