# Supplementary material for: Nitrogen-doped tungsten carbide nanoarray as an efficient bifunctional electrocatalyst for water splitting in acid
Source: Nat Commun. 2018 Mar 2;9:924. doi: 10.1038/s41467-018-03429-z (PMC5834627; doi:10.1038/s41467-018-03429-z)
Supplement: Supplementary file 2 — Description of Additional Supplementary Files [file 41467_2018_3429_MOESM2_ESM.pdf]

Description of Additional Supplementary Files

File Name: Supplementary Movie 1
Description: Video of bubble evolution from N-WC nanoarray, N-WC, WC nanoarray, and WC surface.

File Name: Supplementary Movie 2
Description: |Video of the water splitting process with a N-WC nanoarray cathode and an Ir/C anode at the voltage of 1.5 V without iR correction.

File Name: Supplementary Movie 3
Description: Video of the water splitting process with a N-WC nanoarray cathode and a N-WC nanoarray anode at the voltage of 1.4 V without iR correction.

File Name: Supplementary Movie 4
Description: Video of the water splitting process with a N-WC nanoarray cathode and an Ir/C anode powered by a commercial AA battery.

File Name: Supplementary Movie 5
Description: Video of the water splitting process with a N-WC nanoarray cathode and a N-WC nanoarray anode powered by a commercial AA battery.

File Name: Supplementary Movie 6
Description: Enlarged view of the bubbling process with a N-WC nanoarray cathode and an Ir/C anode powered by a commercial AA battery.

File Name: Supplementary Movie 7
Description: Enlarged view of the bubbling process with a N-WC nanoarray cathode and a N-WC nanoarray anode powered by a commercial AA battery.
